# The Mediating Role of Depression and of State Anxiety οn the Relationship between Trait Anxiety and Fatigue in Nurses during the Pandemic Crisis

**DOI:** 10.3390/healthcare11030367

**Published:** 2023-01-28

**Authors:** Christos Sikaras, Sofia Zyga, Maria Tsironi, Athanasios Tselebis, Argyro Pachi, Ioannis Ilias, Aspasia Panagiotou

**Affiliations:** 1 Nursing Department, “Sotiria” General Hospital of Thoracic Diseases, 11527 Athens, Greece; 2 Department of Nursing, University of Peloponnese, 22100 Tripoli, Greece; 3 Psychiatric Department, “Sotiria” General Hospital of Chest Diseases, 11527 Athens, Greece; 4 Department of Endocrinology, “Elena Venizelou” Hospital, 11521 Athens, Greece

**Keywords:** depression, anxiety, fatigue, COVID-19, nurses, mediation

## Abstract

The coronavirus pandemic (COVID-19) is a global health crisis with a particular emotional and physical impact on health professionals, especially nurses. The aim of this study was to investigate the prevalence of anxiety, depression and fatigue and their possible relationships among nurses during the pandemic. The study population consisted of nurses from five tertiary-level public hospitals in Athens who completed the Fatigue Assessment Scale (FAS), Beck Depression Inventory (BDI) and State–Trait Anxiety Inventory (STAI) questionnaires. Gender, age and years of work experience were recorded. The study was conducted from mid-November to mid-December 2021. The sample included 404 nurses (69 males and 335 females) with a mean age of 42.88 years (SD = 10.90) and 17.96 (SD = 12.00) years of work experience. Symptoms of fatigue were noted in 60.4% of participants, while 39.7% had symptoms of depression, 60.1% had abnormal scores on state anxiety and 46.8% on trait anxiety, with females showing higher scores on all scales (*p* < 0.05). High positive correlations (*p* < 0.01) were found between the FAS, BDI, State Anxiety and Trait Anxiety scales. Regression analysis showed that 51.7% of the variance in FAS scores can be explained by trait anxiety, an additional 6.2% by the BDI and 1.2% by state anxiety. Mediation analysis showed that state anxiety and BDI mediate the relationship between trait anxiety and FAS. Finally, BDI was found to exert a moderating role in the relationship between trait anxiety and fatigue. In conclusion, our study showed that nurses continue to experience high rates of anxiety, depression and fatigue. The variation in fatigue appears to be significantly dependent on trait anxiety. Depressive symptomatology and state anxiety exert a parallel positive mediation on the relationship between trait anxiety and fatigue, with depression exhibiting a moderating role in this relationship.

## 1. Introduction

The 2019 coronavirus pandemic (COVID-19) has caused a major health crisis worldwide with a huge psychological impact [1,2,3]. Regarding health professionals, there is consensus across the literature that they are at increased risk of high stress, anxiety, depression, sleep disorders, burnout and post-traumatic stress disorder, with particular emotional and physical impacts [4,5,6,7,8]. In addition, due to the disruption of the balance between their professional and social lives and the occupational risks associated with exposure to the virus, there is an increase in both physical and mental fatigue [9]. Recent studies have shown that nursing staff have higher rates of emotional symptoms compared to other health professionals [10,11]. Work-related fatigue in nurses has been identified as a threat to their health, but it is also associated with negative consequences for safe and quality patient care [12,13]. Work-related fatigue is a complex and multidimensional condition with emotional, physiological, cognitive, mental and sensory components arising as a consequence of excessive work demands and inadequate energy recovery [13]. Moreover, it is positively correlated with levels of anxiety and depression [12]. Anxiety is one of the most common psychiatric disorders in the general population. It consists of a complex cognitive, emotional, physiological and behavioral response related to preparation for anticipated events or circumstances perceived as threatening [14]. Depression is a common mental disorder that presents with a depressed mood, a loss of interest or pleasure, decreased energy, fatigue, feelings of guilt, sleep or appetite disturbances and a lack of concentration [15,16].

Earlier studies had suggested [17,18] that high levels of trait anxiety were a significant risk factor for the development (onset, severity and outcome) of depression. These findings and the high comorbidity between anxiety and depression (up to 60%), but especially the fact that anxiety disorders precede depressive disorders [19], led Sandi and Richter-Levin [20] to hypothesize a “neurocognitive model”. According to this model, the neurocognitive style of trait anxiety (neurocognitive maladjustments) plays a central role in the pathological development of depression [20]. More recent studies have argued that high trait anxiety is an important vulnerability phenotype for stress-induced depression [21]. Moreover, studies in breast cancer patients report trait anxiety as an important determinant of both depressive symptoms and fatigue [22,23]. Cognitive theory argues that stressful life events activate some stable underlying dysfunctional maladaptive self-schemata of individuals, which, through automatic cognitive processes, lead to the onset or worsening of depression [24,25,26]. In summary, trait anxiety makes an individual susceptible to depression [25].

From the above, it is clear that nursing staff are particularly vulnerable to developing physical and psychological problems, especially during the COVID-19 pandemic. It is worth noting that at the time of the study, Greece was experiencing the peak of the fourth wave of the pandemic with the prevalence of the Delta variant, which caused particularly high mortality rates. At that time (mid-November to mid-December 2021), approximately six to seven thousand new cases were being recorded daily, 600–700 patients were hospitalized in intensive care units and 80-100 deaths per day were attributed to COVID-19, with an increasing trend that was difficult to address by the national health system [27], which suffers from a severe shortage of nursing staff [28]. Therefore, the aim of the present study was to study the prevalence of anxiety, depression and fatigue and to investigate the possible relationships among them. By selecting and implementing targeted interventions aimed at enhancing key protective factors, the development of adverse physical and mental conditions can be harnessed.

## 2. Materials and Methods

### 2.1. Research Design and Procedure

This was a descriptive correlation study. Data were collected through self-completed questionnaires that were distributed in person to the participants by the researchers. Participation in the study was voluntary. The questionnaire was anonymous, participants had the right to voluntarily withdraw from the study and were aware of the objectives and procedures to be followed.

The study population consisted of a convenience sample of 404 nurses from five large public/academic hospitals in Athens. The above hospitals treated patients with and without COVID-19. The demographics of the study participants included gender and age. Professional information included years of work experience. In the invitation to nurses to participate in the study, an effort was made to make the study sample representative of Greek nurses in terms of gender, years of work and age. The Ethics Committee of the University of Peloponnese approved the study protocol (18699/11-10-2021), as did the Clinical Research Committees of the five hospitals. The study was conducted from mid-November to mid-December 2021.

### 2.2. Study Participants

With a target population of 27,103 nurses [29] a margin of error of 5%, a confidence level of 95%, and a percentage of the sample selecting a particular response of 50%, the minimum required sample for the study was set at 379. A total of 404 nurses agreed to participate in the study out of 500 nurses who were asked to answer the questionnaires.

### 2.3. Measures and Instruments

The Fatigue Assessment Scale (FAS) was used to assess fatigue. The FAS consists of 10 questions (e.g., “Fatigue bothers me”). Each question is scored from 1 to 5. Responses include “never, sometimes, often, quite often, always”. Total scores range from 10 to 50, with values ≥22 indicating fatigue. The FAS questions aim to capture fatigue over the few weeks prior to the questionnaire completion [30]. The reliability and validity of the Greek version of the Fatigue Assessment Scale (FAS) have been tested in a Greek population [21]. The scale has been used in studies of nursing staff in Greece [29]. The internal consistency, as indicated by Cronbach’s alpha, was 0.761 [31].

The Beck Depression Inventory (BDI) was used to assess depression. This scale measures the cognitive, emotional, behavioral and physical manifestations of depression in the individual during the week prior to the inventory completion. It consists of 21 items, which are rated on a scale of 0–3 [32]. The items include the following: sadness, pessimism, a feeling of failure, anhedonia, guilt, the expectation of punishment, self-loathing, suicidal ideation, crying, irritability, social withdrawal, indecisiveness, body image, ability to work, insomnia, easy fatigue, anorexia, weight loss, physical preoccupation and loss of libido. The total score is obtained after summing up the scores of the 21 items. The stratification of depressive symptom severity is as follows: 0–9 = no depression, 10–15 = mild depression, 16–23 = moderate depression and ≥24 = severe depression. The scale, in its Greek version [33], is a brief and reliable tool for measuring depression and has been applied to nursing staff in Greece [34]. Its internal consistency and reliability are high, and retest reliability ranges from 0.48–0.86 in clinical settings and 0.60–0.90 in the non-clinical population. Validity with respect to an external criterion for depression, such as a clinical diagnosis, is considered satisfactory [33].

The Spielberger State–Trait Anxiety Inventory (STAI-Y Form) was used to assess anxiety. This scale consists of forty items, each of which is scored from 1 to 4. The scale differentiates between anxiety caused by a specific situation (state anxiety) and anxiety that is a more permanent personality trait (trait anxiety). The State Anxiety subscale (STAI Form Y-1) consists of 20 items that assess how the respondent is feeling “right now”. In responding to the State Anxiety subscale, individuals select the response that best describes the intensity of their feelings. Answers include “Not at all, somewhat, moderately and a lot”. The Trait Anxiety subscale (STAI Form Y-2) consists of 20 items that assess how the respondent feels “in general”. In the Trait Anxiety subscale, individuals rate the frequency of their feelings. Answers include “almost never, sometimes, often and almost always”. Scores for each subscale can vary from a minimum of 20 to a maximum of 80. Higher scores indicate more anxiety [35]. The scale, in its Greek form, is a short and reliable tool for measuring anxiety. Its internal consistency (Cronbach’s alpha) was 0.93 for the State Anxiety subscale and 0.92 for the Trait Anxiety subscale. It is considered to have high internal consistency, reliability and validity [36,37].

### 2.4. Data Analysis

All variables were evaluated using descriptive statistics, and values were expressed as means and standard deviations for continuous variables. The prevalence of fatigue, anxiety and depression was determined as a percentage. Independent t-tests were conducted to assess continuous variables according to gender. Pearson’s correlation was performed to determine the strength and direction of the relationship between variables. Linear regression models were constructed to investigate whether the associated variables were significant predictors of the independent variable. The linear regression hypotheses (linear relationship, independence, homoscedasticity and normality) were assessed by visual inspection of the variables, residual plots and quantile–quantile (QQ) plots. Statistical significance was set at *p* < 0.05 (two-tailed) and analyses were performed using IBM SPSS Statistics 23 (IBM SPSS Statistics for Windows, Version 23.0, IBM Corp, Armonk, NY, USA). Mediation and moderation analyses were performed using the Hayes SPSS Process Macro Models 4 and 5.

## 3. Results

The sample consisted of 404 nurses (69 men and 335 women) with a mean age of 42.88 years (SD = 10.90) and 17.96 (SD = 12.00) years of experience as nurses (Table 1). In terms of gender (x^2^), years of work and age (sample t-test), the sample showed no statistically significant difference (*p* > 0.05) compared to representative samples of the country’s nursing workforce from other studies [29].

Descriptive statistics of the scales are presented in Table 1. Female nurses showed higher means in all scales compared to the male population (Table 1).

The percentage of nurses who showed fatigue (FAS ≥ 22) was 60.4%. The mean fatigue (FAS: 24.08) was statistically lower (sample t-test *p* < 0.01) compared to the mean fatigue (FAS: mean = 25.61, SD = 7.37, N = 701) experienced by nurses in the previous pandemic wave [29]. However, by calculating Hedges’ g between the previous measurement and the present measurement, we found a small effect size (g = 0.208).

Regarding depression, 39.7% of the participants had symptoms of depression, (22.4% mild symptoms, 13.7% moderate and 3.6% severe). The mean depressive symptomatology (BDI: 8.94) was shown to be statistically lower (sample t-test *p* < 0.01) compared to the mean depressive symptomatology (BDI: mean = 10.62, SD = 7.65, N = 660) experienced by nurses in a previous phase of the pandemic [34]. By calculating Hedges’ g in this instance, we also found a small effect size (g = 0.222).

A total of 60.1% of the participants were found to have abnormal scores in state anxiety and 46.8% in trait anxiety. High positive correlations (*p* < 0.01, Table 2) were found between the fatigue, depression, state anxiety and trait anxiety scales. Age showed a negative correlation with trait anxiety (*p* < 0.05) and a positive correlation (*p* < 0.01) with years of work (Table 2).

A stepwise multiple regression analysis was performed to identify the best predictors of fatigue. With fatigue as the dependent variable, gender, age, years of work, depression, Spielberger Trait Anxiety and Spielberger State Anxiety were given as independent variables. This regression showed that 51.7% of the variance in the Fatigue Assessment Scale score could be explained through the Spielberger Trait Anxiety Inventory, an additional 6.2% was explained through the Beck Depression Inventory and 1.2% through the Spielberger State Anxiety Inventory (Table 3). The other variables did not explain the variance in the Fatigue Assessment Scale.

With the Hayes SPSS Process Macro (Model 4 with parallel mediators Beck Depression Inventory and Spielberger State Anxiety) bootstrapping was performed in order to first examine whether depression mediates the relationship between trait anxiety and fatigue and, secondly, in the same way, whether state anxiety mediates the relationship between trait anxiety and fatigue.

**Figure 1:**
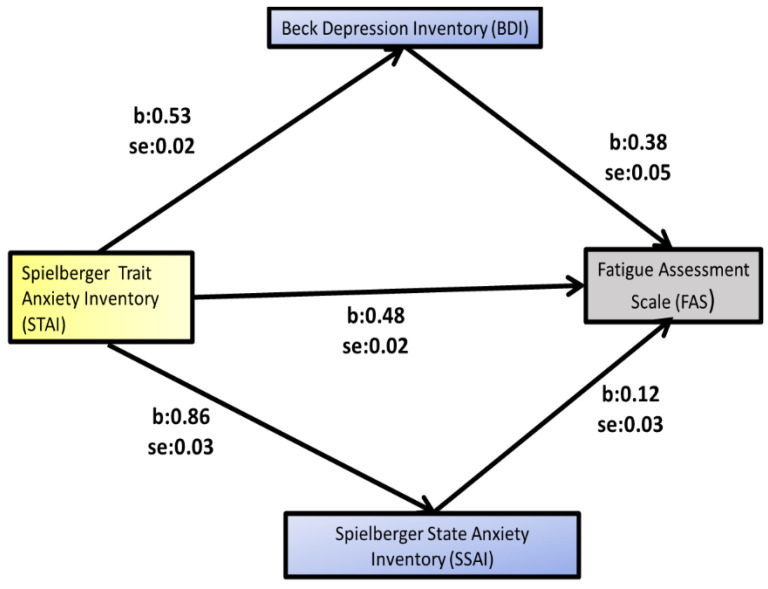
Mediation analysis of Beck Depression Inventory and Spielberger State Anxiety Inventory on Spielberger Trait Anxiety Inventory–Fatigue Assessment Scale relationship (Model 4 with multiple mediators).

Based on 5000 bootstrap samples, a significant indirect relationship between trait anxiety and fatigue was found to be mediated by depressive symptomatology (Table 4, Figure 1).

The mediator variable for the analysis was the Beck Depression Inventory score. The outcome variable for the analysis was the Fatigue Assessment Scale score. The predictor variable for the analysis was the Trait Anxiety Inventory with the variables gender, work experience and age as covariates. The indirect effect of BDI on fatigue was found to be statistically significant [B = 0.1968, 95% CI (0.1368, 0.2587), *p* < 0.05] (Table 4, Figure 1). The mediation proportion of the model for depression was 41%.

In the same analysis, we used Spielberger State Anxiety as a mediator variable; as an outcome variable, the Fatigue Assessment Scale; as a predictor variable, the Spielberger Trait Anxiety Inventory; and the variables gender, work experience and age as covariates. The indirect effect of Spielberger State Anxiety on fatigue was found to be statistically significant [B = 0.0950, 95% CI (0.0446, 0.1471), *p* < 0.05] (Table 4, Figure 1). The mediation proportion of the model for state anxiety was 20%.

Finally, we examined the moderating role of both the Spielberger State Anxiety and depression in the Spielberger Trait Anxiety–fatigue relationship. Spielberger State Anxiety did not show a moderating role.

In contrast, depression emerged as a positive moderator. Specifically, moderation analysis was performed using the PROCESS method model 5 (with the Spielberger Trait Anxiety Inventory as the predictor variable, the Fatigue Assessment Scale as the outcome variable and the Beck Depression Inventory as the moderator variable). Depression showed a statistically significant moderating role in the Spielberger Trait Anxiety Inventory and Fatigue Assessment Scale relationship (*p* < 0.01, Table 5).

**Figure 2:**
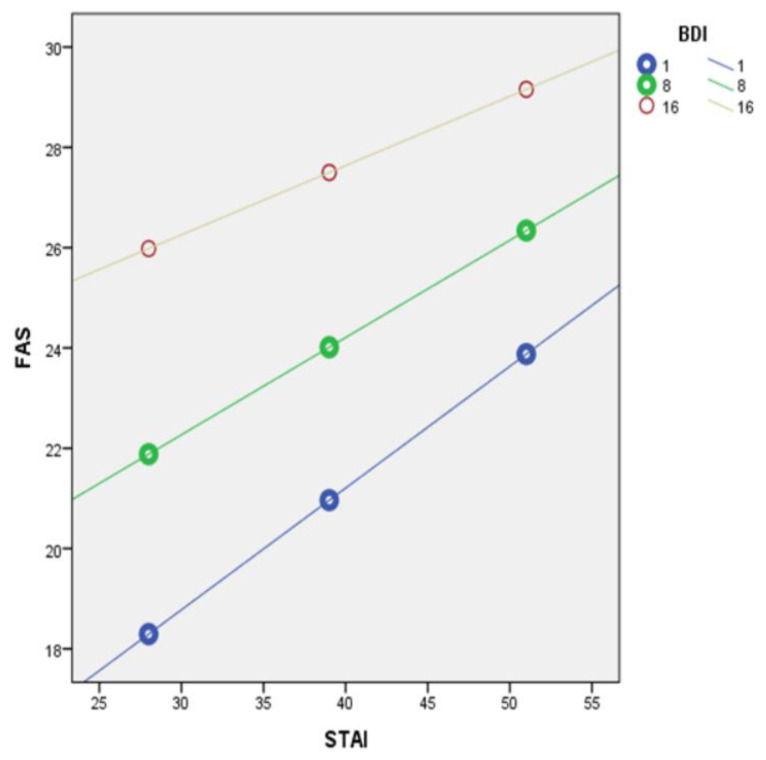
Beck Depression Inventory (BDI) as a moderator of the relationship between Spielberger Trait Anxiety Inventory (STAI) and Fatigue Assessment Scale (FAS), at low (1), middle (8) and high (16) degrees of BDI.

At low moderation (BDI = 1) the conditional effect was 0.2428 [95% CI (0.1512, 0.3343), *p* < 0.05]. At medium moderation (BDI = 8) the conditional effect was 0.1939 [95% CI (0.1096, 0.2783), *p* < 0.05]. At high moderation (BDI = 16) the conditional effect was 0.1381 [95% CI (0.0475, 0.2287), *p* < 0.05] (Figure 2).

## 4. Discussion

Alongside the COVID-19 pandemic, an emerging health problem is very likely to co-exist. This problem is pandemic fatigue (PF), which, according to the World Health Organization (WHO), is defined as the physical and mental fatigue that can occur during a pandemic as a consequence of changes in a person’s usual activities due to the various measures implemented to reduce the transmission of the virus [38].

Nurses are particularly vulnerable to developing mental and psychological problems, such as higher rates of anxiety, depression, fatigue, mental distress, emotional exhaustion and post-traumatic stress disorder during the peak of the pandemic, potentially increasing the risk of developing PF [39].

Consistent with the above, the results of the present study showed a high prevalence of fatigue, situational and structural anxiety and depression (60.4%, 60.1%, 46.8% and 39.7%, respectively). Our results also showed that Greek nurses had among the highest rates of fatigue [9,12,39], anxiety and depression compared to the findings of studies in other countries [6,10,11].

These findings are probably due, among other reasons, to the fact that Greece, during the period of this study, was experiencing the worst phase of the pandemic [27] in terms of the number of hospitalized patients and mortality, and the resilience of the national health system due to the pandemic was put to test. In addition, it is worth noting that Greece has the lowest ratio of nurses per 1000 inhabitants among European Union countries [28], a finding that partly justifies these findings. Literature data support the positive role of adequate nurse staffing in reducing fatigue, stress and physical and mental exhaustion due to better workload management [39] and subsequent perceived control over work [40]. On the other hand, the resolution of serious problems—such as the provision of personal protective equipment that occurred in the first year of the pandemic [29], the familiarization of nurses with the disease, the increase in information and knowledge about it, and, above all, the complete and universal vaccination of the health care workers’ population—are factors that may have been beneficial and may account for the reduction in depression and fatigue compared to the first year of the pandemic. It should be stressed here that this reduction, although statistically significant, does not appear to be clinically important, but it is a finding that may make us more optimistic for the future.

It is known that the prevalence of anxiety and depression is almost twice as high in women as in men [41,42,43,44,45], and studies report that this ratio begins to equalize in the general population with increasing age [46]. In nursing staff, a consistent finding is that the female population has higher rates of anxiety and depression than male nurses [37]. The negative correlation of age with anxiety in the study population confirms the decrease in symptoms of anxiety brought about with increasing age. This negative correlation is well-known among Greek nurses [37]. Studies have tried to explain the increased presence of depressive and anxiety symptomatology by proposing both hormonal factors [46] and social factors [47]. It is possible that both social and instrumental factors are responsible for the presence of increased fatigue in female nurses compared to male nurses.

Statistically significant correlations were found among all the scales considered. Anxiety (state–trait), depression and chronic fatigue were positively correlated with each other. Numerous studies confirm the association between anxiety, depression and fatigue in both nurses [48] and patients with chronic diseases [49]. The findings of this study support a model where trait anxiety accounts for a large proportion of the variance in fatigue.

Furthermore, depressive symptomatology and state anxiety are aggravatingly mediated by depressive symptomatology. This dual mediation works in a parallel fashion, although the mediation effect of depression is twice that of state anxiety. Depressive symptoms are important in this population as they act as a moderator in the relationship between trait anxiety and fatigue. This effect was observed at high, medium and low levels of depression. According to a study by Polikandrioti et al., both anxiety and depression can affect fatigue levels in different ways [50]. People with anxiety are more vulnerable to panic, fear and other high-stress reactions that sequentially increase fatigue levels. In addition, individuals with depression lack motivation or energy to perform either physical or mental tasks and often experience changes in sleep patterns, which in turn increase fatigue levels [50]. However, the association between fatigue and anxiety or depression appears to be a vicious cycle, as a reduction in one can dramatically reduce the risk of developing the other [49], while the presence of one can dramatically increase the risk of developing the other [50].

Ultimately, the nature, as well as the direction, of causality between these variables remains uncertain. While depression appears to be the stronger mediator of the effect of trait anxiety on fatigue, the relationship between the two mediators remains unclear. In future research, it will be useful to use the four-way decomposition method for greater clarity. In addition, our study was synchronic and could not determine with certainty the cause-and-effect relationship beyond the creation of a model. Despite the recent increase in research interest in this area, more longitudinal and/or intervention studies that could further enrich our understanding of the anxiety–fatigue–depression relationship are desirable.

The prevalence of anxiety, depression and fatigue seems to be very high among nurses in Greek hospitals. To reduce the psychological impact of the COVID-19 pandemic on nurses, a number of studies and meta-analyses highlight the positive role of organizational support [51], interventions focused on enhancing resilience [52], appropriate nurse-to-patient ratios [39], proper work time management [53], appropriate remuneration [54], developing fatigue risk management systems (FRMS) [53,55] enhancing a sense of coherence [34] and strengthening family support [56,57]. On the other hand, the use of anxiolytic and antidepressant drugs seems to have increased in the community [58,59] without it being clear what is happening among health professionals. A limitation of the study is that it did not examine the above factors as possible confounding variables.

We have to note that the role of night work was not examined. Nurses working in rotating shifts or at night are exposed to sleep disturbances due to abnormal melatonin secretion at night and circadian rhythm disturbance [60,61]. Nurses are at a high risk of fatigue due to stressful work environments with heavy workloads and non-standard work schedules [62]. Stress and emotional distress are known to predict sleep quality, and all of these factors have been shown to predict fatigue severity [63].

Considering the higher prevalence of insomnia in female nurses compared to other health professionals [64,65] and the fact that they constitute the vast majority of nursing staff, it is proposed to strengthen health protection in this most vulnerable category through prevention and intervention programs oriented towards their psychosocial support [65].

Finally, it is important to mention the willingness of nurses to participate in this research. It is common for Greek nurses to show high rates of acceptance to participate in studies with questionnaires, even in studies conducted online [29]. Nevertheless, the motivation of nurses’ acceptance in studies with questionnaires has not been adequately investigated, although it is particularly important as it may influence the prevalence rates of the variables.

## 5. Conclusions

In the second year of the pandemic, nursing staff continue to experience high rates of anxiety, depression and fatigue. These findings are probably due, among other reasons, to the fact that Greece, during the period of this study, was experiencing the worst phase of the pandemic. The variation in fatigue appears to be significantly dependent on trait anxiety. Depressive symptomatology and state anxiety exert parallel positive mediation on the trait anxiety and fatigue relationship. In this relationship, depression has a moderating role at low, medium and high values. Despite the recent increase in research interest in this area, more longitudinal and/or intervention studies that could further enrich our understanding of the anxiety–fatigue–depression relationship are desirable. Finally, it is proposed to strengthen health protection in this most vulnerable category through prevention and intervention programs oriented toward nurses’ psychosocial support.

## Figures and Tables

**Table 1:** General characteristics of nursing staff and fatigue/anxiety/depression scores with regards to gender.

Participants	Descriptive Statistics	Age	Work Experience (in Years)	Fatigue Assessment Scale	State Anxiety Inventory	Trait Anxiety Inventory	Beck Depression Inventory
Male N = 69	Mean	41.16	15.60	21.25 **	35.47 **	36.32 **	7.06 *
	SD	11.37	11.67	7.43	12.14	11.35	7.15
Female N = 335	Mean	43.23	18.45	24.66 **	40.18 **	40.30 **	9.33 *
	SD	10.79	12.02	7.20	11.59	10.52	7.48
Total N = 404	Mean	42.88	17.96	24.08	39.38	39.62	8.94
	SD	10.90	12.00	7.35	11.80	10.76	7.46

Notes: * independent t-test *p* < 0.05; ** independent t-test *p* < 0.01.

**Table 2:** Correlations among age, work experience (in years), fatigue, anxiety and depression.

Pearson Correlation N = 404		AGE	Work Experience (in Years)	Fatigue Assessment Scale	State Anxiety Inventory	Trait Anxiety Inventory
Work Experience (in Years)	r	0.885 **				
	*p*	0.001				
Fatigue Assessment Scale	r	−0.096	−0.082			
	*p*	0.055	0.104			
Spielberger State Anxiety Inventory	r	−0.056	−0.06	0.635 **		
	*p*	0.258	0.232	0.001		
Spielberger Trait Anxiety Inventory	r	−0.101 *	−0.094	0.715 **	0.789 **	
	*p*	0.043	0.064	0.001	0.001	
Beck Depression Inventory	r	0.003	0.001	0.707 **	0.603 **	0.750 **
	*p*	0.951	0.991	0.001	0.001	0.001

* Correlation is significant at the 0.05 level (two-tailed). ** Correlation is significant at the 0.01 level (two-tailed).

**Table 3:** Stepwise multiple regression (only statistically significant variables are included).

Dependent Variable: Fatigue Assessment Scale	R Square	R Square Change	Beta	*t*	*p*	Durbin-Watson
Spielberger Trait Anxiety Inventory	0.517	0.517	0.297	4.68	0.01 *	1.945
Beck Depression Inventory	0.577	0.062	0.373	7.58	0.01 *	
Spielberger State Anxiety Inventory	0.591	0.012	0.180	3.43	0.01 *	

Notes: Beta = standardized regression coefficient; correlations are statistically significant at the * *p* < 0.01 level.

**Table 4:** Mediation analysis of Beck Depression Inventory (BDI) and Spielberger State Anxiety Inventory (SSAI) on Spielberger Trait Anxiety Inventory (STAI)–Fatigue Assessment Scale (FAS) relationship *.

Variable		b	SE	*t*	*p*	95% Confidence Interval	
						LLCI	ULCI
STAI → BDI		0.5256	0.0233	22.5438	0.001	0.4798	0.5715
STAI → SSAI		0.8575	0.0347	24.7243	0.001	0.7993	0.9257
STAI → BDI → FAS		0.3744	0.0484	7.7436	0.001	0.2794	0.4695
STAI → SSAI → FAS		0.1107	0.0325	3.4066	0.001	0.0468	0.1747
STAI → FAS		0.4803	0.0242	19.8655	0.001	0.4237	0.5278
Effects							
Direct		0.1885	0.0433	4.3516	0.001	0.1033	0.2736
Indirect **	Total	0.2918	0.0399			0.2158	0.3689
	BDI	0.1968	0.0311			0.1368	0.2587
	SSAI	0.095	0.0262			0.0446	0.1471
Total (STAI → FAS)		0.4803	0.0242	19.8655	0.001	0.4327	0.5278

* Gender, work experience and age were included in the analysis as covariates variables. They are not shown in the table as they did not give significant statistical results (*p* > 0.05). ** Based on 5000 bootstrap samples.

**Table 5:** Moderation analysis: Beck Depression Inventory (BDI) as a moderator of the relationship between Spielberger Trait Anxiety Inventory (STAI) and Fatigue Assessment Scale (FAS) with Spielberger State Anxiety as a mediator *.

Outcome Variable: Fatigue Assessment Scale (FAS)	b	SE	*t*	*p*
Constant	7.3902 [3.1924, 11.5880]	2.1351	3.4614	0.01
Spielberger Trait Anxiety Inventory (STAI)	0.2498 [0.1563, 0.3432]	0.0475	5.2561	0.01
Beck Depression Inventory (BDI)	0.7079 [0.4694, 0.9465]	0.1213	5.834	0.01
Interaction(STAI × BDI)	−0.0070 [−0.0116, −0.0024]	0.0023	−2.9908	0.01

* Gender, work experience and age were included in the analysis as covariate variables. They are not shown in the table as they did not give significant statistical results (*p* > 0.05).

## Data Availability

The data and the questionnaires of the study are available upon request from the corresponding author.
